# Bis{4-(all­yloxy)-*N*′-[4-(oct­yloxy)benzyl­idene]benzohydrazidato}nickel(II)

**DOI:** 10.1107/S2414314625001208

**Published:** 2025-02-14

**Authors:** Sultana Shakila Khan, Md. Belayet Hossain Howlader, Md. Chanmiya Sheikh, Ryuta Miyatake, Ennio Zangrando

**Affiliations:** a Department of Pharmacy, Pabna University of Science and Technology, Pabna-6600, Bangladesh; b Department of Chemistry, Rajshahi University, Rajshahi-6205, Bangladesh; c Division of Applied Chemistry Graduate School of Natural Science and Technology Okayama University, 1-1 Tsushima-naka 3-Chome Okayama 700-8530 Japan; d Center for Environmental Conservation and Research Safety University of Toyama, 3190 Gofuku Toyama 930-8555 Japan; e Department of Chemical and Pharmaceutical Sciences, University of Trieste, Italy; University of Antofagasta, Chile

**Keywords:** crystal structure, benzohydrazide ligand, Ni^II^ complex, *trans* configuration complex, octyl alkyl chain

## Abstract

The central Ni^II^ atom in the title complex, [Ni(C_25_H_31_N_2_O_3_)_2_], is located on crystallographic inversion center and adopts a slightly distorted square-planar coordination geometry defined by the imine nitro­gen and amide oxygen donors of two chelating symmetry-related ligands in a *trans* configuration. The Ni—N and Ni—O bond lengths are 1.8685 (15) and 1.8409 (14) Å, respectively, with a chelating N—Ni—O bond angle of 83.79 (6)°. These data are in agreement with those determined for complexes of similar ligands.

## Abstract 2

A bis­chelated mononuclear nickel(II) complex with benzohydrazide ligands bearing an all­yloxy group and a long saturated alkyl chain is reported.

## Graphical abstract

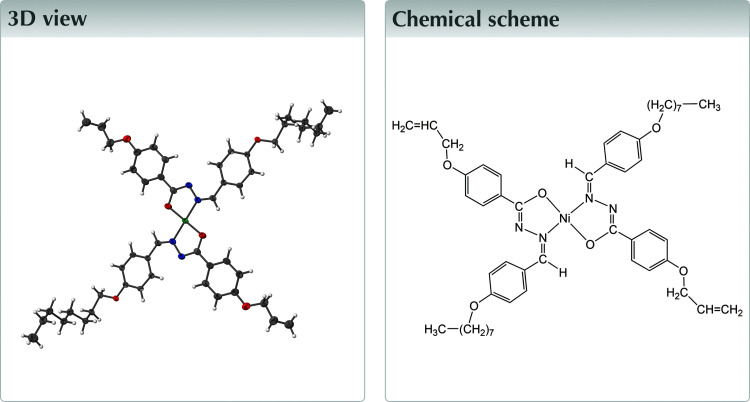

## Structure description

**Figure 1:**
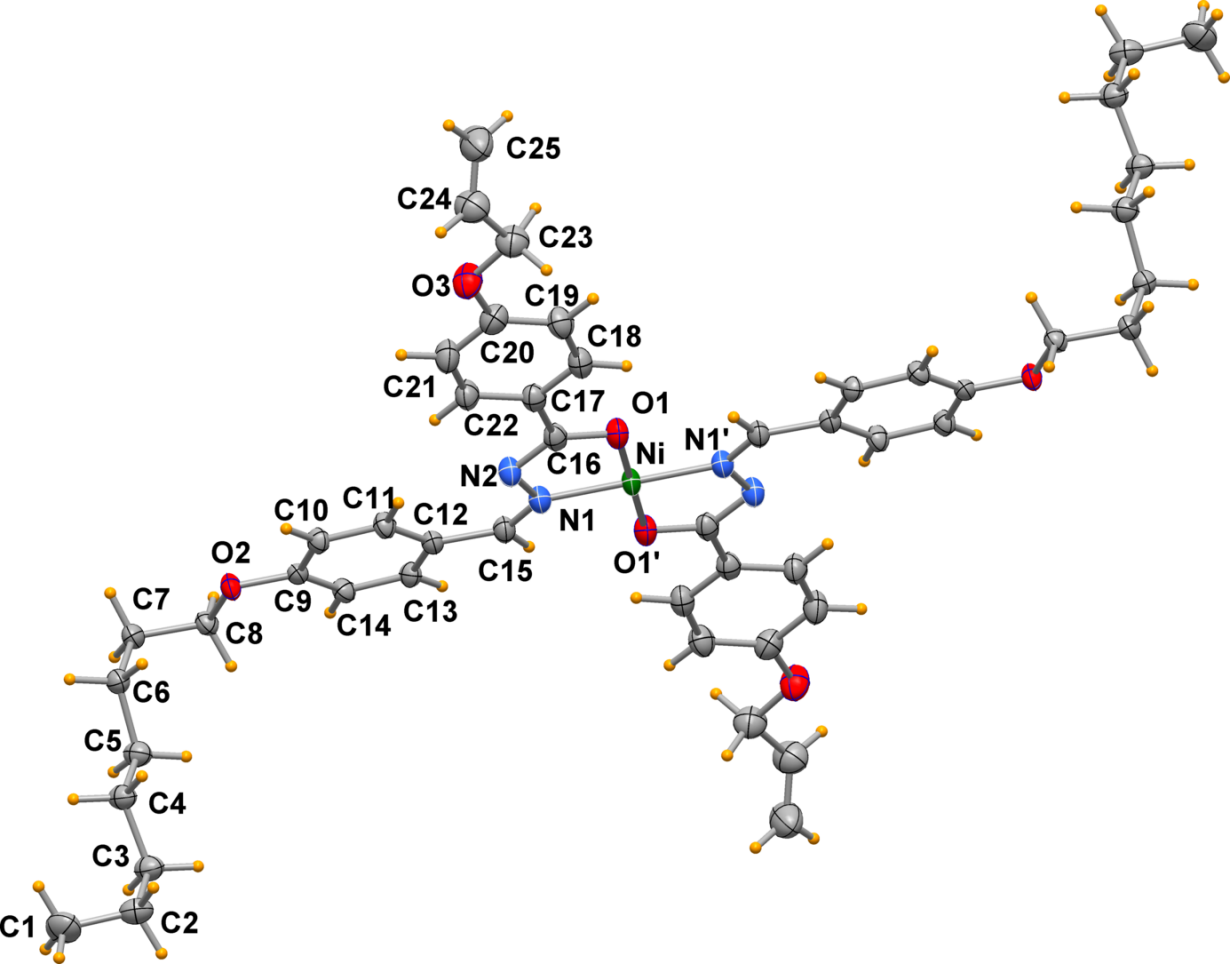
The title complex (displacement ellipsoid probability at 50%) showing the atom-labeling scheme for the asymmetric unit. [Symmetry code: (′) 2 − *x*, 1 − *y*, 2 − *z*.]

The title compound (Fig. 1 ▸) crystallizes in the triclinic system, space group *P*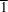, with the nickel(II) atom located on a center of symmetry, thus the asymmetric unit comprises half of the mol­ecule and the *trans* configuration of donor atoms is imposed by the crystal symmetry. The Ni—O1 and Ni—N1 bond lengths are 1.8409 (14) and 1.8685 (15) Å, respectively, and the chelating O—Ni—N angle is of 83.78 (6)°. With the exception of the all­yloxy fragment (atoms O3 and C23–25) and the oct­yloxy (atoms O2 and C1–C8) chain, all atoms of the chelating benzohydrazide ligands and of phenyl group C9–14 are almost coplanar (max displacement of ±0.1 Å), indicating an extended electron delocalization about the central Ni atom. The allyl C24—C25 bond length is 1.292 (4) Å. The octyl alkyl chains adopt a staggered conformation for the C2–C7 chain, while the chain is kinked at both ends with C6—C7—C8—O2 and C1—C2—C3—C4 torsion angles of −64.7 (2) and 68.1 (2)°, respectively, likely for packing requirements. Bond lengths and angles around the central metal atom are in close agreement with values determined in similar complexes with a square-planar coordination environment (Banna *et al.* 2022 ▸, 2024 ▸; Al-Qadsy *et al.* 2021 ▸; Mondal *et al.*, 2014 ▸; Neethu *et al.* 2021 ▸), indicating that the steric and electronic properties of the different groups bound to the hydrazone ligands have no appreciable influence on the central metal atom.

**Figure 2:**
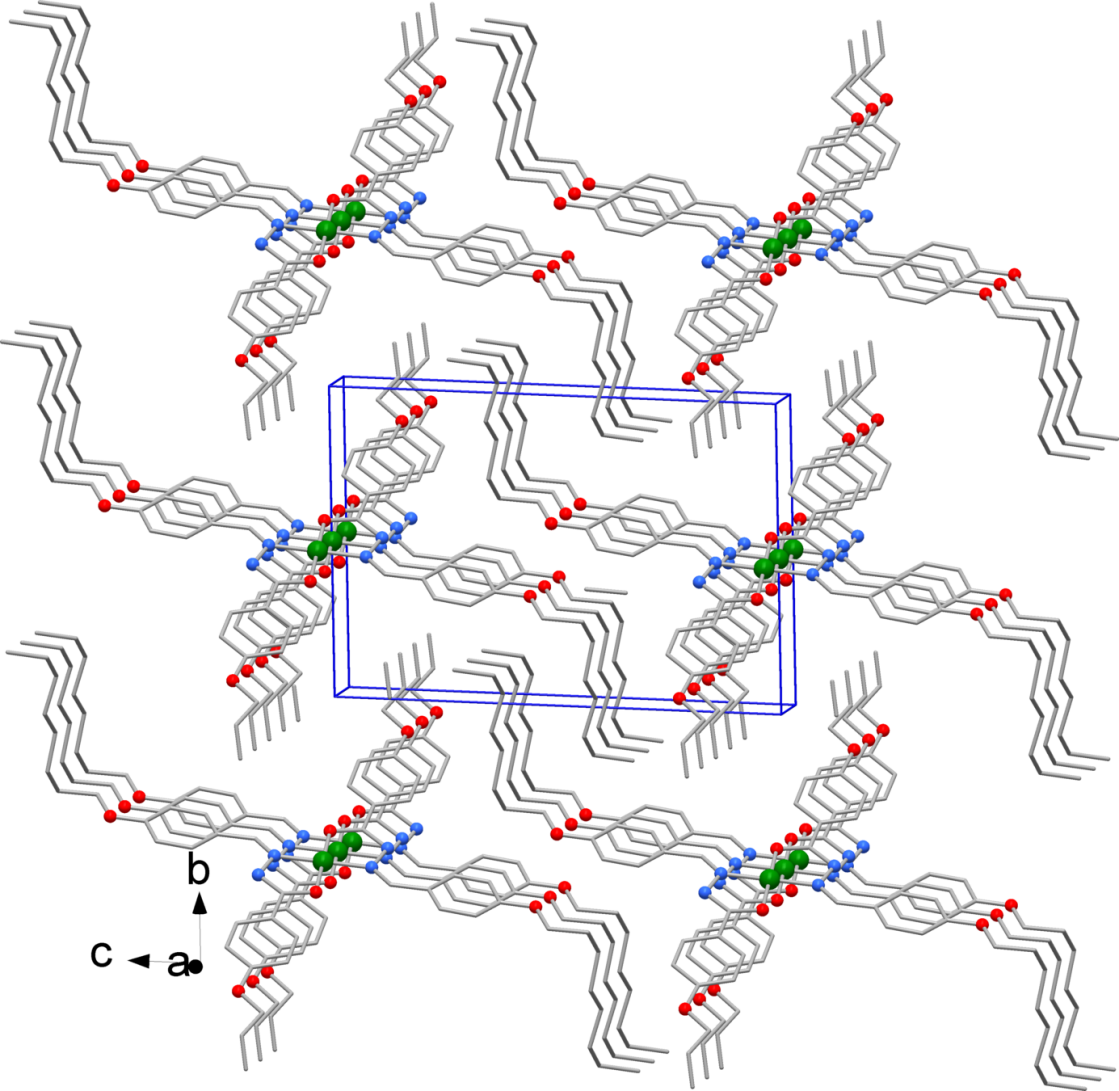
Crystal packing viewed down the *a* axis. (H atoms not shown for clarity.)

**Figure 3:**
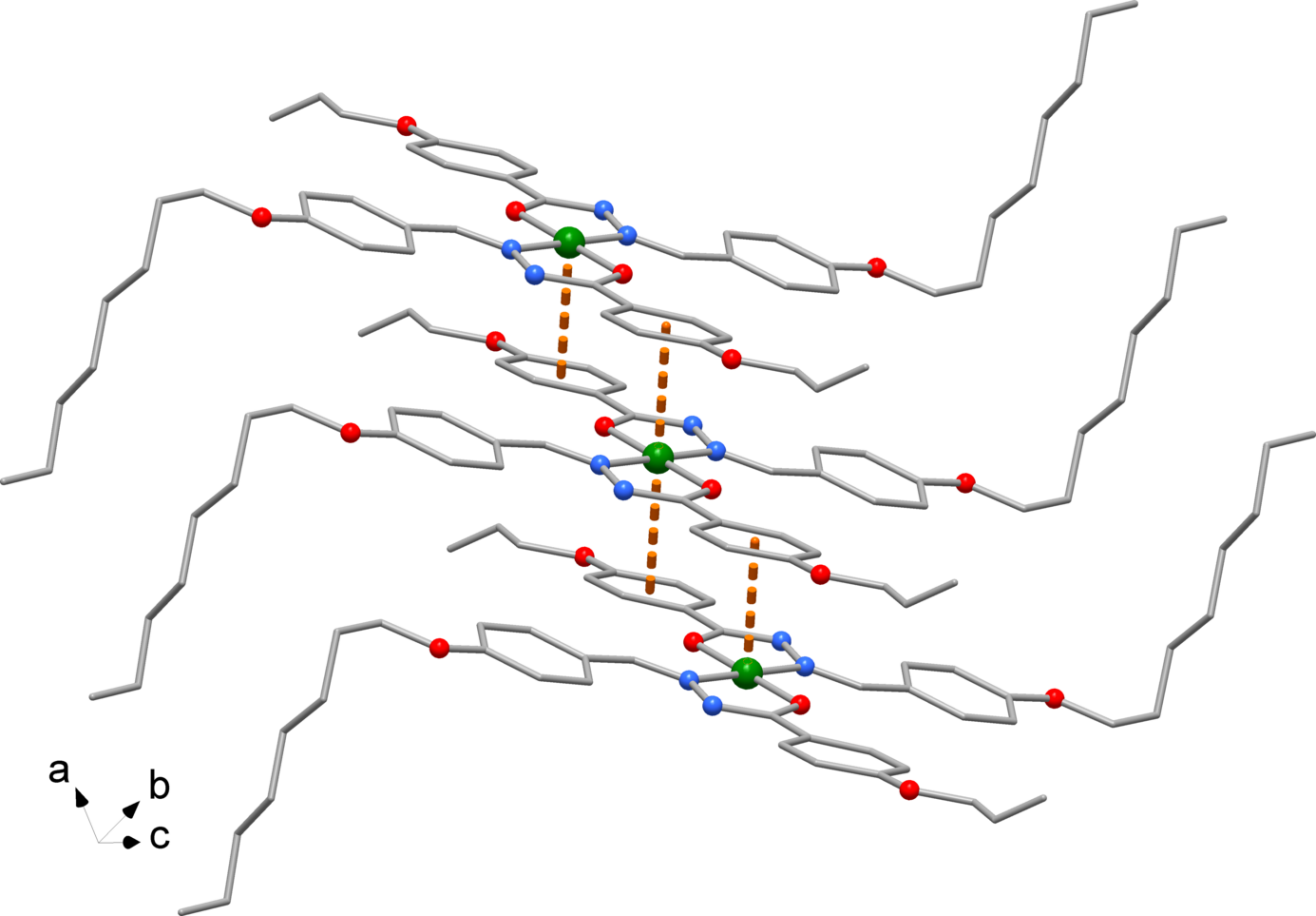
Detail of the crystal packing showing the Ni atom sandwiched by phenyl rings.

The crystal packing viewed down the *a* axis is shown in Fig. 2 ▸. It is worth noting the metal atom is sandwiched by the phenyl rings of symmetry-related complexes indicating Ni⋯π-ring inter­actions between the complexes with an Ni–ring centroid distance of 3.688 Å (Fig. 3 ▸).

## Synthesis and crystallization

An ethano­lic solution (20 ml) of 4-(all­yloxy)benzoyl­hydrazine (0.501 g, 2.6 mmol) was added to 4-octyloxybenzaldehyde (0.609 g, 2.6 mmol) dissolved in ethanol (10 ml). The resulting mixture was refluxed for 1 h. Subsequently, a solution of nickel(II) acetate tetra­hydrate (0.326 g, 1.3 mmol in 10 ml of ethanol) was introduced, and refluxing was continued for an additional 3 h. This led to the formation of an orange precipitate that was separated by filtration and washed with hot ethanol. Orange single crystals suitable for X-ray analysis were obtained by gradual evaporation from a chloro­form and aceto­nitrile mixture (1:1, *v*/*v*) over a period of 21 d, followed by filtration and drying under vacuum in a desiccator containing anhydrous CaCl_2_.

Orange crystal, yield: 0.85 g, 75%, m p. = 143°C.

IR data (KBr disc, cm^−1^): 1598 ν(C=N), 1499 ν(C=C), 1021 ν(N—N), 588 ν(*M*—N), 509 ν(*M*—O).

^1^H NMR (CDCl_3_, 400 MHz), δ: 8.31 (*d*, 2×2H, C-6, 8, *J* = 9.2 Hz), 7.92 (*d*, 2×2H, C-13, 17, *J* = 9.2 Hz), 7.12 (*s*, 2×1H, C-11, –CH=N,), 6.96 (*d*, 2×2H, C-5, 9, *J* = 9.2), 6.90 (*d*, 2×2H, C-14, 16, *J* = 9.2 Hz,), 6.12–6.02 (*m*, 2×1H, C-2, H_c_), 5.44 (*dq*, 2×1H, C-1, H_a_), 5.32 (*dq*, 2×1H, C-1, H_b_), 4.59 (*d*, 2×2H, C-3, –OCH_2_), 4.03 (*t*, 2×2H, C-18, OCH_2_), 1.83 (*p*, 2×2H, C-19), 1.47(*p*, 2×2H, C-20), 1.40–1.25 (*m*, 2×8H, C-21, 22, 23, 24), 0.89(*t*, 2×3H, C-25, CH_3_).

UV-Vis spectrum in CHCl_3_ [λ_max_ nm, ɛ_max_*M*^−1^cm^−1^)]: 265 (25660), 288 (31660), 322 (35000), 361 (22340), 394 (20920), 411 (20280).

## Refinement

Crystal data, data collection and structure refinement are summarized in Table 1 ▸.

## Supplementary Material

Crystal structure: contains datablock(s) global, I. DOI: 10.1107/S2414314625001208/bx4033sup1.cif

Structure factors: contains datablock(s) I. DOI: 10.1107/S2414314625001208/bx4033Isup2.hkl

CCDC reference: 2335127

Additional supporting information:  crystallographic information; 3D view; checkCIF report

## Figures and Tables

**Table 1:** Experimental details

Crystal data	
Chemical formula	[Ni(C_25_H_31_N_2_O_3_)_2_]
*M*_r_	873.74
Crystal system, space group	Triclinic, *P* 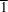
Temperature (K)	173
*a*, *b*, *c* (Å)	5.5532 (3), 12.0594 (5), 17.0230 (8)
α, β, γ (°)	86.599 (6), 83.723 (6), 82.426 (6)
*V* (Å^3^)	1122.13 (9)
*Z*	1
Radiation type	Mo *K*α
μ (mm^−1^)	0.49
Crystal size (mm)	0.14 × 0.07 × 0.02

Data collection	
Diffractometer	Rigaku R-AXIS RAPID
Absorption correction	Multi-scan (*ABSCOR*; Rigaku, 1995 ▸)
*T*_min_, *T*_max_	0.840, 0.990
No. of measured, independent and observed [*I* > 2σ(*I*)] reflections	10908, 5100, 4014
*R*_int_	0.033
(sin θ/λ)_max_ (Å^−1^)	0.649

Refinement	
*R*[*F*^2^ > 2σ(*F*^2^)], *wR*(*F*^2^), *S*	0.044, 0.094, 1.02
No. of reflections	5100
No. of parameters	278
H-atom treatment	H-atom parameters constrained
Δρ_max_, Δρ_min_ (e Å^−3^)	0.39, −0.26

Computer programs: *RAPID-AUTO* (Rigaku, 2010 ▸), *SHELXT2014/5* (Sheldrick, 2015*a* ▸), *SHELXL2019/2* (Sheldrick, 2015*b* ▸) and *CrystalStructure* (Rigaku, 2019 ▸.

## full crystallographic data

### Bis(N'-{[4-(octyloxy)phenyl]methylidene}-4-(prop-2-en-1-yloxy)benzohydrazidato)nickel(II) Crystal data

**Table:**

[Ni(C_25_H_31_N_2_O_3_)_2_]	*Z* = 1
*M**_r_* = 873.74	*F*(000) = 466
Triclinic, *P*1	*D*_x_ = 1.293 Mg m^−^^3^
*a* = 5.5532 (3) Å	Mo *K*α radiation, λ = 0.71075 Å
*b* = 12.0594 (5) Å	Cell parameters from 8635 reflections
*c* = 17.0230 (8) Å	θ = 2.0–27.5°
α = 86.599 (6)°	µ = 0.49 mm^−^^1^
β = 83.723 (6)°	*T* = 173 K
γ = 82.426 (6)°	Platelet, orange
*V* = 1122.13 (9) Å^3^	0.14 × 0.07 × 0.02 mm

### Bis(N'-{[4-(octyloxy)phenyl]methylidene}-4-(prop-2-en-1-yloxy)benzohydrazidato)nickel(II) Data collection

**Table:**

Rigaku R-AXIS RAPID diffractometer	4014 reflections with *I* > 2σ(*I*)
ω scans	*R*_int_ = 0.033
Absorption correction: multi-scan (ABSCOR; Rigaku, 1995)	θ_max_ = 27.5°, θ_min_ = 2.9°
*T*_min_ = 0.840, *T*_max_ = 0.990	*h* = −7→6
10908 measured reflections	*k* = −15→15
5100 independent reflections	*l* = −22→22

### Bis(N'-{[4-(octyloxy)phenyl]methylidene}-4-(prop-2-en-1-yloxy)benzohydrazidato)nickel(II) Refinement

**Table:**

Refinement on *F*^2^	0 restraints
Least-squares matrix: full	Hydrogen site location: inferred from neighbouring sites
*R*[*F*^2^ > 2σ(*F*^2^)] = 0.044	H-atom parameters constrained
*wR*(*F*^2^) = 0.094	*w* = 1/[σ^2^(*F*_o_^2^) + (0.0373*P*)^2^ + 0.4947*P*] where *P* = (*F*_o_^2^ + 2*F*_c_^2^)/3
*S* = 1.02	(Δ/σ)_max_ < 0.001
5100 reflections	Δρ_max_ = 0.39 e Å^−^^3^
278 parameters	Δρ_min_ = −0.26 e Å^−^^3^

### Bis(N'-{[4-(octyloxy)phenyl]methylidene}-4-(prop-2-en-1-yloxy)benzohydrazidato)nickel(II) Special details

**Table:**

Geometry. All esds (except the esd in the dihedral angle between two l.s. planes) are estimated using the full covariance matrix. The cell esds are taken into account individually in the estimation of esds in distances, angles and torsion angles; correlations between esds in cell parameters are only used when they are defined by crystal symmetry. An approximate (isotropic) treatment of cell esds is used for estimating esds involving l.s. planes.

### Bis(N'-{[4-(octyloxy)phenyl]methylidene}-4-(prop-2-en-1-yloxy)benzohydrazidato)nickel(II) Fractional atomic coordinates and isotropic or equivalent isotropic displacement parameters (Å^2^)

**Table:**

	*x*	*y*	*z*	*U*_iso_*/*U*_eq_	
Ni1	1.000000	0.500000	1.000000	0.02555 (11)	
O2	1.3156 (2)	0.39178 (11)	0.51487 (7)	0.0272 (3)	
O1	0.7075 (2)	0.58957 (11)	0.99238 (7)	0.0313 (3)	
O3	−0.1756 (3)	0.88993 (14)	0.84389 (9)	0.0459 (4)	
N1	0.9936 (3)	0.48735 (14)	0.89141 (9)	0.0289 (4)	
N2	0.7837 (3)	0.54396 (14)	0.86172 (9)	0.0305 (4)	
C1	0.9657 (5)	−0.1354 (2)	0.30337 (15)	0.0539 (7)	
H1A	0.924174	−0.061646	0.277505	0.065*	
H1B	0.836194	−0.181922	0.298985	0.065*	
H1C	1.120435	−0.171450	0.277660	0.065*	
C2	0.9909 (4)	−0.12196 (17)	0.38963 (13)	0.0377 (5)	
H2A	1.016646	−0.197144	0.416317	0.045*	
H2B	0.836226	−0.082433	0.414423	0.045*	
C3	1.1995 (4)	−0.05776 (16)	0.40339 (12)	0.0348 (5)	
H3A	1.351869	−0.093339	0.374511	0.042*	
H3B	1.221544	−0.063783	0.460479	0.042*	
C4	1.1605 (4)	0.06573 (16)	0.37706 (12)	0.0315 (4)	
H4A	1.144580	0.071857	0.319556	0.038*	
H4B	1.005242	0.100830	0.404511	0.038*	
C5	1.3653 (4)	0.13003 (16)	0.39362 (12)	0.0323 (4)	
H5A	1.516692	0.101308	0.360912	0.039*	
H5B	1.395144	0.116517	0.449873	0.039*	
C6	1.3093 (4)	0.25547 (15)	0.37622 (11)	0.0279 (4)	
H6A	1.156858	0.283582	0.408611	0.033*	
H6B	1.279765	0.268496	0.319913	0.033*	
C7	1.5106 (4)	0.32294 (16)	0.39263 (10)	0.0276 (4)	
H7A	1.667812	0.288387	0.366078	0.033*	
H7B	1.476372	0.399690	0.369053	0.033*	
C8	1.5370 (3)	0.33040 (16)	0.47970 (10)	0.0263 (4)	
H8A	1.562562	0.254437	0.505230	0.032*	
H8B	1.678896	0.369471	0.486457	0.032*	
C9	1.2890 (3)	0.40152 (14)	0.59504 (10)	0.0224 (4)	
C10	1.0664 (3)	0.45783 (16)	0.62585 (10)	0.0256 (4)	
H10	0.947015	0.486275	0.591372	0.031*	
C11	1.0180 (3)	0.47257 (16)	0.70626 (10)	0.0262 (4)	
H11	0.865513	0.510709	0.726758	0.031*	
C12	1.1944 (3)	0.43120 (15)	0.75792 (10)	0.0237 (4)	
C13	1.4157 (3)	0.37686 (15)	0.72535 (10)	0.0256 (4)	
H13	1.536885	0.349306	0.759374	0.031*	
C14	1.4657 (3)	0.36145 (15)	0.64485 (10)	0.0246 (4)	
H14	1.618655	0.323997	0.624127	0.030*	
C15	1.1673 (4)	0.44005 (16)	0.84378 (10)	0.0281 (4)	
H15	1.303146	0.404296	0.869019	0.034*	
C16	0.6494 (4)	0.59609 (17)	0.92044 (11)	0.0291 (4)	
C17	0.4257 (4)	0.67020 (17)	0.90249 (11)	0.0294 (4)	
C18	0.2732 (4)	0.72326 (18)	0.96231 (11)	0.0334 (5)	
H18	0.308620	0.708848	1.015579	0.040*	
C19	0.0678 (4)	0.79769 (18)	0.94600 (12)	0.0367 (5)	
H19	−0.034176	0.834476	0.987607	0.044*	
C20	0.0150 (4)	0.81708 (18)	0.86844 (12)	0.0361 (5)	
C21	0.1636 (4)	0.76076 (19)	0.80798 (12)	0.0399 (5)	
H21	0.124748	0.772476	0.754906	0.048*	
C22	0.3656 (4)	0.68858 (18)	0.82496 (11)	0.0352 (5)	
H22	0.465618	0.650708	0.783432	0.042*	
C23	−0.3349 (4)	0.9511 (2)	0.90235 (14)	0.0443 (5)	
H23A	−0.243037	0.999510	0.929629	0.053*	
H23B	−0.408270	0.898833	0.942242	0.053*	
C24	−0.5296 (5)	1.0207 (2)	0.86110 (15)	0.0498 (6)	
H24	−0.606909	0.985027	0.824224	0.060*	
C25	−0.6015 (6)	1.1256 (2)	0.87151 (16)	0.0678 (8)	
H25A	−0.528832	1.164291	0.907875	0.081*	
H25B	−0.727128	1.164038	0.842807	0.081*	

### Bis(N'-{[4-(octyloxy)phenyl]methylidene}-4-(prop-2-en-1-yloxy)benzohydrazidato)nickel(II) Atomic displacement parameters (Å^2^)

**Table:**

	*U*^11^	*U*^22^	*U*^33^	*U*^12^	*U*^13^	*U*^23^
Ni1	0.0277 (2)	0.0329 (2)	0.01594 (16)	−0.00290 (15)	−0.00241 (13)	−0.00230 (14)
O2	0.0301 (7)	0.0322 (7)	0.0180 (6)	0.0033 (6)	−0.0029 (5)	−0.0045 (5)
O1	0.0323 (8)	0.0399 (8)	0.0221 (6)	−0.0045 (6)	−0.0045 (5)	−0.0012 (6)
O3	0.0411 (9)	0.0540 (10)	0.0380 (8)	0.0101 (8)	−0.0050 (7)	0.0018 (7)
N1	0.0307 (9)	0.0336 (9)	0.0222 (7)	−0.0033 (7)	−0.0020 (6)	−0.0025 (7)
N2	0.0291 (9)	0.0388 (9)	0.0229 (8)	−0.0003 (7)	−0.0041 (7)	−0.0026 (7)
C1	0.0678 (18)	0.0453 (14)	0.0559 (15)	−0.0210 (13)	−0.0212 (13)	−0.0040 (12)
C2	0.0370 (12)	0.0282 (11)	0.0485 (12)	−0.0054 (9)	−0.0053 (10)	−0.0006 (9)
C3	0.0386 (12)	0.0266 (10)	0.0400 (11)	−0.0033 (9)	−0.0100 (9)	0.0001 (9)
C4	0.0338 (11)	0.0254 (10)	0.0369 (10)	−0.0034 (8)	−0.0109 (9)	−0.0009 (8)
C5	0.0340 (11)	0.0276 (10)	0.0363 (10)	−0.0032 (9)	−0.0089 (9)	−0.0025 (8)
C6	0.0336 (11)	0.0255 (10)	0.0248 (9)	−0.0029 (8)	−0.0056 (8)	−0.0018 (8)
C7	0.0353 (11)	0.0257 (10)	0.0216 (8)	−0.0039 (8)	−0.0012 (8)	−0.0035 (7)
C8	0.0285 (10)	0.0265 (9)	0.0234 (8)	−0.0003 (8)	−0.0011 (7)	−0.0053 (7)
C9	0.0288 (10)	0.0203 (9)	0.0187 (8)	−0.0056 (7)	−0.0019 (7)	−0.0014 (7)
C10	0.0263 (10)	0.0288 (10)	0.0223 (8)	−0.0017 (8)	−0.0069 (7)	−0.0014 (7)
C11	0.0248 (10)	0.0300 (10)	0.0228 (8)	0.0010 (8)	−0.0020 (7)	−0.0036 (7)
C12	0.0271 (10)	0.0246 (9)	0.0199 (8)	−0.0040 (8)	−0.0027 (7)	−0.0021 (7)
C13	0.0276 (10)	0.0266 (9)	0.0233 (8)	−0.0022 (8)	−0.0083 (7)	−0.0005 (7)
C14	0.0239 (10)	0.0252 (9)	0.0245 (8)	−0.0012 (8)	−0.0022 (7)	−0.0040 (7)
C15	0.0325 (11)	0.0316 (10)	0.0207 (8)	−0.0051 (9)	−0.0040 (8)	−0.0014 (8)
C16	0.0305 (11)	0.0340 (10)	0.0234 (9)	−0.0090 (8)	0.0003 (8)	−0.0019 (8)
C17	0.0295 (10)	0.0338 (11)	0.0255 (9)	−0.0077 (8)	−0.0017 (8)	−0.0015 (8)
C18	0.0348 (12)	0.0421 (12)	0.0237 (9)	−0.0071 (9)	−0.0021 (8)	−0.0025 (8)
C19	0.0347 (12)	0.0432 (12)	0.0309 (10)	−0.0025 (10)	0.0019 (9)	−0.0054 (9)
C20	0.0344 (12)	0.0383 (12)	0.0345 (10)	−0.0041 (9)	−0.0029 (9)	0.0043 (9)
C21	0.0429 (13)	0.0491 (13)	0.0260 (10)	0.0004 (10)	−0.0061 (9)	0.0010 (9)
C22	0.0396 (12)	0.0408 (12)	0.0236 (9)	−0.0016 (10)	−0.0001 (8)	−0.0013 (8)
C23	0.0389 (13)	0.0448 (13)	0.0468 (13)	0.0031 (10)	−0.0034 (10)	−0.0023 (11)
C24	0.0450 (14)	0.0481 (14)	0.0562 (15)	0.0022 (11)	−0.0129 (12)	−0.0047 (12)
C25	0.092 (2)	0.0566 (17)	0.0520 (15)	0.0152 (16)	−0.0234 (15)	−0.0039 (13)

### Bis(N'-{[4-(octyloxy)phenyl]methylidene}-4-(prop-2-en-1-yloxy)benzohydrazidato)nickel(II) Geometric parameters (Å, º)

**Table:**

Ni1—O1	1.8409 (14)	C7—H7B	0.9900
Ni1—O1^i^	1.8410 (14)	C8—H8A	0.9900
Ni1—N1	1.8685 (15)	C8—H8B	0.9900
Ni1—N1^i^	1.8685 (15)	C9—C14	1.387 (2)
O2—C9	1.3669 (19)	C9—C10	1.394 (3)
O2—C8	1.439 (2)	C10—C11	1.383 (2)
O1—C16	1.295 (2)	C10—H10	0.9500
O3—C20	1.369 (3)	C11—C12	1.410 (2)
O3—C23	1.428 (3)	C11—H11	0.9500
N1—C15	1.286 (2)	C12—C13	1.389 (3)
N1—N2	1.399 (2)	C12—C15	1.462 (2)
N2—C16	1.318 (2)	C13—C14	1.387 (2)
C1—C2	1.510 (3)	C13—H13	0.9500
C1—H1A	0.9800	C14—H14	0.9500
C1—H1B	0.9800	C15—H15	0.9500
C1—H1C	0.9800	C16—C17	1.481 (3)
C2—C3	1.521 (3)	C17—C18	1.382 (3)
C2—H2A	0.9900	C17—C22	1.394 (3)
C2—H2B	0.9900	C18—C19	1.398 (3)
C3—C4	1.524 (3)	C18—H18	0.9500
C3—H3A	0.9900	C19—C20	1.383 (3)
C3—H3B	0.9900	C19—H19	0.9500
C4—C5	1.518 (3)	C20—C21	1.397 (3)
C4—H4A	0.9900	C21—C22	1.372 (3)
C4—H4B	0.9900	C21—H21	0.9500
C5—C6	1.522 (3)	C22—H22	0.9500
C5—H5A	0.9900	C23—C24	1.491 (3)
C5—H5B	0.9900	C23—H23A	0.9900
C6—C7	1.525 (3)	C23—H23B	0.9900
C6—H6A	0.9900	C24—C25	1.292 (4)
C6—H6B	0.9900	C24—H24	0.9500
C7—C8	1.514 (2)	C25—H25A	0.9500
C7—H7A	0.9900	C25—H25B	0.9500
			
O1—Ni1—O1^i^	180.00 (8)	O2—C8—H8B	110.2
O1—Ni1—N1	83.79 (6)	C7—C8—H8B	110.2
O1^i^—Ni1—N1	96.21 (6)	H8A—C8—H8B	108.5
O1—Ni1—N1^i^	96.21 (6)	O2—C9—C14	124.71 (16)
O1^i^—Ni1—N1^i^	83.79 (6)	O2—C9—C10	115.20 (15)
N1—Ni1—N1^i^	180.00 (10)	C14—C9—C10	120.08 (16)
C9—O2—C8	118.00 (13)	C11—C10—C9	120.45 (16)
C16—O1—Ni1	110.83 (12)	C11—C10—H10	119.8
C20—O3—C23	118.14 (17)	C9—C10—H10	119.8
C15—N1—N2	119.86 (16)	C10—C11—C12	120.25 (17)
C15—N1—Ni1	126.02 (14)	C10—C11—H11	119.9
N2—N1—Ni1	113.90 (11)	C12—C11—H11	119.9
C16—N2—N1	107.83 (15)	C13—C12—C11	117.96 (16)
C2—C1—H1A	109.5	C13—C12—C15	115.99 (16)
C2—C1—H1B	109.5	C11—C12—C15	126.05 (17)
H1A—C1—H1B	109.5	C14—C13—C12	122.23 (16)
C2—C1—H1C	109.5	C14—C13—H13	118.9
H1A—C1—H1C	109.5	C12—C13—H13	118.9
H1B—C1—H1C	109.5	C9—C14—C13	119.02 (17)
C1—C2—C3	113.89 (19)	C9—C14—H14	120.5
C1—C2—H2A	108.8	C13—C14—H14	120.5
C3—C2—H2A	108.8	N1—C15—C12	131.82 (18)
C1—C2—H2B	108.8	N1—C15—H15	114.1
C3—C2—H2B	108.8	C12—C15—H15	114.1
H2A—C2—H2B	107.7	O1—C16—N2	123.60 (18)
C2—C3—C4	113.93 (17)	O1—C16—C17	118.14 (16)
C2—C3—H3A	108.8	N2—C16—C17	118.20 (16)
C4—C3—H3A	108.8	C18—C17—C22	118.66 (19)
C2—C3—H3B	108.8	C18—C17—C16	120.56 (17)
C4—C3—H3B	108.8	C22—C17—C16	120.77 (17)
H3A—C3—H3B	107.7	C17—C18—C19	121.30 (18)
C5—C4—C3	113.58 (16)	C17—C18—H18	119.3
C5—C4—H4A	108.9	C19—C18—H18	119.3
C3—C4—H4A	108.9	C20—C19—C18	119.10 (19)
C5—C4—H4B	108.9	C20—C19—H19	120.4
C3—C4—H4B	108.9	C18—C19—H19	120.4
H4A—C4—H4B	107.7	O3—C20—C19	125.21 (19)
C4—C5—C6	112.82 (16)	O3—C20—C21	114.91 (18)
C4—C5—H5A	109.0	C19—C20—C21	119.9 (2)
C6—C5—H5A	109.0	C22—C21—C20	120.26 (19)
C4—C5—H5B	109.0	C22—C21—H21	119.9
C6—C5—H5B	109.0	C20—C21—H21	119.9
H5A—C5—H5B	107.8	C21—C22—C17	120.74 (19)
C5—C6—C7	114.30 (16)	C21—C22—H22	119.6
C5—C6—H6A	108.7	C17—C22—H22	119.6
C7—C6—H6A	108.7	O3—C23—C24	107.57 (19)
C5—C6—H6B	108.7	O3—C23—H23A	110.2
C7—C6—H6B	108.7	C24—C23—H23A	110.2
H6A—C6—H6B	107.6	O3—C23—H23B	110.2
C8—C7—C6	113.99 (16)	C24—C23—H23B	110.2
C8—C7—H7A	108.8	H23A—C23—H23B	108.5
C6—C7—H7A	108.8	C25—C24—C23	125.2 (2)
C8—C7—H7B	108.8	C25—C24—H24	117.4
C6—C7—H7B	108.8	C23—C24—H24	117.4
H7A—C7—H7B	107.7	C24—C25—H25A	120.0
O2—C8—C7	107.42 (15)	C24—C25—H25B	120.0
O2—C8—H8A	110.2	H25A—C25—H25B	120.0
C7—C8—H8A	110.2		
			
N1—Ni1—O1—C16	0.87 (13)	N2—N1—C15—C12	−0.6 (3)
N1^i^—Ni1—O1—C16	−179.13 (13)	Ni1—N1—C15—C12	−174.90 (16)
O1—Ni1—N1—C15	172.55 (18)	C13—C12—C15—N1	177.3 (2)
O1^i^—Ni1—N1—C15	−7.45 (18)	C11—C12—C15—N1	−2.6 (3)
O1—Ni1—N1—N2	−2.05 (13)	Ni1—O1—C16—N2	0.6 (2)
O1^i^—Ni1—N1—N2	177.95 (13)	Ni1—O1—C16—C17	−176.57 (14)
C15—N1—N2—C16	−172.28 (18)	N1—N2—C16—O1	−2.2 (3)
Ni1—N1—N2—C16	2.7 (2)	N1—N2—C16—C17	174.98 (16)
C1—C2—C3—C4	68.1 (2)	O1—C16—C17—C18	−5.3 (3)
C2—C3—C4—C5	177.99 (17)	N2—C16—C17—C18	177.36 (19)
C3—C4—C5—C6	−173.03 (17)	O1—C16—C17—C22	174.15 (19)
C4—C5—C6—C7	179.67 (16)	N2—C16—C17—C22	−3.2 (3)
C5—C6—C7—C8	−70.8 (2)	C22—C17—C18—C19	−2.7 (3)
C9—O2—C8—C7	174.85 (15)	C16—C17—C18—C19	176.84 (19)
C6—C7—C8—O2	−64.7 (2)	C17—C18—C19—C20	1.0 (3)
C8—O2—C9—C14	3.5 (3)	C23—O3—C20—C19	−0.3 (3)
C8—O2—C9—C10	−177.39 (15)	C23—O3—C20—C21	−179.7 (2)
O2—C9—C10—C11	179.80 (17)	C18—C19—C20—O3	−178.1 (2)
C14—C9—C10—C11	−1.1 (3)	C18—C19—C20—C21	1.3 (3)
C9—C10—C11—C12	0.3 (3)	O3—C20—C21—C22	177.7 (2)
C10—C11—C12—C13	0.6 (3)	C19—C20—C21—C22	−1.8 (3)
C10—C11—C12—C15	−179.54 (18)	C20—C21—C22—C17	0.0 (3)
C11—C12—C13—C14	−0.7 (3)	C18—C17—C22—C21	2.1 (3)
C15—C12—C13—C14	179.38 (17)	C16—C17—C22—C21	−177.4 (2)
O2—C9—C14—C13	179.96 (17)	C20—O3—C23—C24	−178.13 (19)
C10—C9—C14—C13	0.9 (3)	O3—C23—C24—C25	−132.8 (3)
C12—C13—C14—C9	0.0 (3)		

Symmetry code: (i) −*x*+2, −*y*+1, −*z*+2.
